# Measurements of Microstructural, Chemical, Optical, and Electrical Properties of Silicon-Oxygen-Nitrogen Films Prepared by Plasma-Enhanced Atomic Layer Deposition

**DOI:** 10.3390/nano8121008

**Published:** 2018-12-05

**Authors:** Hong-Ping Ma, Hong-Liang Lu, Jia-He Yang, Xiao-Xi Li, Tao Wang, Wei Huang, Guang-Jie Yuan, Fadei F. Komarov, David Wei Zhang

**Affiliations:** 1 State Key Laboratory of ASIC and System, Shanghai Institute of Intelligent Electronics & Systems, School of Microelectronics, Fudan University, Shanghai 200433, China; hpma@fudan.edu.cn (H.-P.M.); 18212020044@fudan.edu.cn (J.-H.Y.); 18212020023@fudan.edu.cn (X.-X.L.); 15110720069@fudan.edu.cn (T.W.); dwzhang@fudan.edu.cn (D.W.Z.); 2 SMIT Center, School of Automation and Mechanical Engineering, Shanghai University, Shanghai 201800, China; guangjie@shu.edu.cn; 3 A.N. Sevchenko Institute of Applied Physical Problems, Belarusian State University, 220045 Minsk, Belarus; komarovf@bsu.by

**Keywords:** silicon nitride, silicon oxynitride, plasma enhanced atomic layer deposition, oxygen contamination, optical properties

## Abstract

In this study, silicon nitride (SiN_x_) thin films with different oxygen concentration (i.e., SiON film) were precisely deposited by plasma enhanced atomic layer deposition on Si (100) substrates. Thus, the effect of oxygen concentration on film properties is able to be comparatively studied and various valuable results are obtained. In detail, x-ray reflectivity, x-ray photoelectron spectroscopy, atomic force microscopy, and spectroscopic ellipsometry are used to systematically characterize the microstructural, optical, and electrical properties of SiON film. The experimental results indicate that the surface roughness increases from 0.13 to 0.2 nm as the oxygen concentration decreases. The refractive index of the SiON film reveals an increase from 1.55 to 1.86 with decreasing oxygen concentration. Accordingly, the band-gap energy of these films determined by oxygen 1s-peak analysis decreases from 6.2 to 4.8 eV. Moreover, the I-V tests demonstrate that the film exhibits lower leakage current and better insulation for higher oxygen concentration in film. These results indicate that oxygen affects microstructural, optical, and electrical properties of the prepared SiN_x_ film.

## 1. Introduction

Silicon (Si)-based photonic components have received more and more attention recently. Their compatibility with conventional microelectronic device fabrication techniques and materials makes them attractive for potential applications in integrated optoelectronic technologies [1]. In the last decade, a variety of Si photonic components, such as optical modulators, optical waveguides, and optical detectors, have been successfully received [2]. However, silicon-based luminescent devices have not yet been achieved and still have a long way to go. Since the discovery of efficient photoluminescence in the red region in porous Si at room temperature [3], semiconductor nanoparticles have received much attention for their outstanding optical properties, especially, research on porous Si and Si nanoparticles indicates sustainable growth over the last decade [4,5,6]. In recent years, silicon nanocrystals (SiNCs) have roused significant attention on the applications of light sources [7] and “all-silicon” devices like silicon lasers, light-emitting diodes (LEDs), flash memories [8], tandem solar cells, and so on. The superlattice structure in a solid matrix allows to produce size-, density-, and shape-controlled SiNCs [9,10,11].

In recent years, a significant number of studies have been reported on SiNCs embedded in silicon dioxide (SiO_2_) and silicon nitride (Si_3_N_4_) [12,13]. In the case of SiNCs in SiO_2_, the correlation between the photoluminescence (PL) properties and the size of the nanocrystals has been discovered according to quantum confinement theory. However, because of the influence induced by the interface states between Si and SiO_2_ and the surface passivation of the nanocrystals with oxygen, the emission energy and luminescence intensity of SiNCs in silicon dioxide are limited [14,15,16]. Thus, buffering the silicon-rich silicon nitride (SRN) by an oxide layer has become an effective way to solve the problem [12]. Because of the excellent quality of the silicon nitride matrix [13], this method can in principle enhance the luminescence properties and ensure the quantum confinement effect [17,18,19].

Atomic layer deposition (ALD) is promising technology for advanced thin film deposition as it offers excellent control at the atomic scale over the thickness and uniformity of the film [20,21,22]. This allows the precise preparation of size- and distribution-controlled silicon nanocrystals. Thus, it will be a wonderful opportunity to fabricate silicon nitride and related superlattice by the ALD technique, and study its photoluminescence properties. Although there are many advantages of using the ALD technique in this area, there is still a lot of challenges that should be captured. First of all, most of the SiNCs and superlattices in previous studies are fabricated by CVD [23,24,25], sputtering [26,27,28], or other deposition techniques [29], there are few researches using ALD to fabricate superlattices. Besides, it is inevitable that there always exist oxygen defects or oxygen concentration in SiN_x_ deposition. Therefore, preparing high quality silicon nitride film by ALD is one of the primary challenges. 

There have been a certain number of studies on the growth of SiN_x_ film using ALD [30,31,32], but rarely on its optical and electrical properties. Specifically, systematic study on the effect of oxygen contamination on photoelectrical properties of SiN_x_ film is not present. Thus, in this study, plasma-enhanced atomic layer deposition was used to observe SiN_x_ film with different content of oxygen by the purpose of studying the effect of oxygen on the physical, chemical, and photoelectrical properties of SiN_x_ film (in other words this should be called SiON film). Specifically, basic film characteristics including microstructure, surface morphology, and roughness were evaluated. Then, attention was focused on the chemical bonding character of the obtained film, particularly the effect of oxygen content on the film’s chemical composition while the variation of binding energy was analyzed by Si 2p and O 1s in detail. Furthermore, optical properties like optical constant and energy band-gap of the SiON film with different oxygen content were determined by XPS measurements. At last, electrical properties of these SiON film were analysed to evaluate the effect of oxygen on electrical breakdown strength of these films and other electrical properties. 

## 2. Experimental Section

### 2.1. Preparation of SiON Film

**Figure 1:**
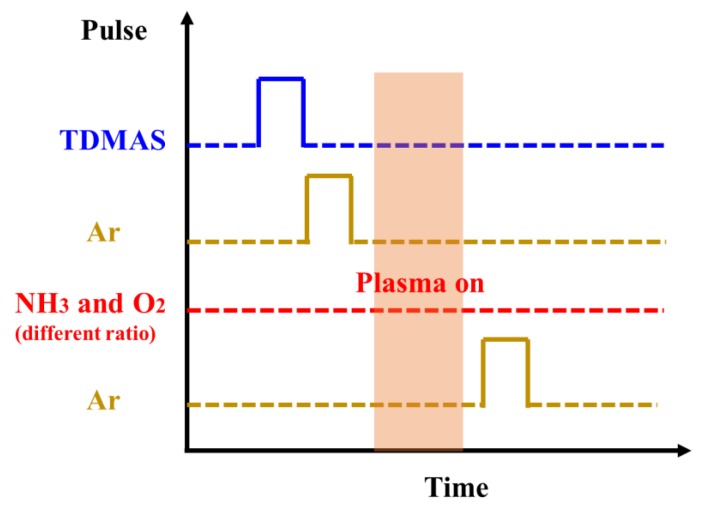
Parameters of one atomic layer deposition (ALD) cycle of the SiON growth utilized in this work.

SiON films with different oxygen concentration were sequentially deposited on p-type (1−10 Ω·cm) and p-type (0.01−1 Ω·cm) single polished Si(100) wafers in a BENEQ TFS200 ALD system (BENEQ, Finland) at 300 °C. The samples grown on p-type (0.01−1 Ω·cm) single polished Si(100) wafers were used to fabricate devices and measure electrical properties. Prior to deposition, the Si wafers were cleaned by a standard RCA process followed by a deionized water rinsing and drying in N_2_. During the deposition process, precursors for Si, N, and O were tris(dimethylamino)silane (TDMAS), N_2_ plasma, and O_2_ plasma, respectively. TDMAS was maintained at 20 °C in a stainless bottle. N_2_ and O_2_ plasma were activated at 200 W. The schematic diagram of one ALD cycle of SiON growth utilized in this work is shown in Figure 1. Each ALD cycle contains four steps: TDMAS pulse (2 s)/Ar purge (2 s)/plasma processing (10 s)/Ar purge (4s). In our experiment, different oxygen contents of SiON film are achieved by varying the flow of oxygen. It should be noted that the N_2_ and the O_2_ are simultaneously led into the chamber with different ratios in this work for realizing the direct growth of the SiON in one ALD cycle. At 300 °C, the O_2_ and the N_2_ react only with TDMAS when the plasma is turned on. As a result, the N_2_/O_2_ mixed gas is passed over the wafer continually during the deposition. For SiON-1 sample, the ratio of N_2_/O_2_ is 90:10, for SiON-2 sample, the ratio of N_2_/O_2_ is 95:5, then for SiON-3 sample, the ratio of N_2_/O_2_ is 99:1.

### 2.2. Sample Characterization

The microstructure and morphology of the films were characterized by X-ray reflection (XRR) (Bruker, D8, Billerica, MA, USA) and glow discharge optical emission spectroscopy (GDOES) (HIOKI, 200D, Nagano-ken, Japan). The surface morphology was observed using an atomic force microscope (AFM, Bruker, icon), and a typical 1 × 1 μm area was investigated using non-contact mode. SE measurements were performed on a rotating analyzer ellipsometer (SOPRA, GES-5E, Annecy, France). The incident angles were 65°, 70°, and 75°. The spectral wavelength range from 190 to 800 nm with a step of 2 nm, the system measured the spectra of Ψ and Δ as functions of wavelength (λ). The resulting spectra was fitted with WinElli_II software. The chemical bonding character of the obtained film was characterised by X-ray photoelectron spectroscopy (XPS) (SPECS, Berlin, Germany) using a monochromatic Al Kα source (hν = 1486.6 eV). A narrow scan resolution of 0.1 eV was used. The adventitious C 1s peak, arising from traces of hydrocarbon in the spectrometer, was used as a reference for evaluating the peak positions because of static charging of samples. The C 1s peak position was observed together with other peaks (Si 2p, N 1s, and O 1s) of the spectrum, and all the XPS spectra were calibrated by the C 1s peak at a binding energy of 284.6 eV. 

## 3. Results and Discussion

### 3.1. Surface Morphology and Microstructure

**Figure 2:**
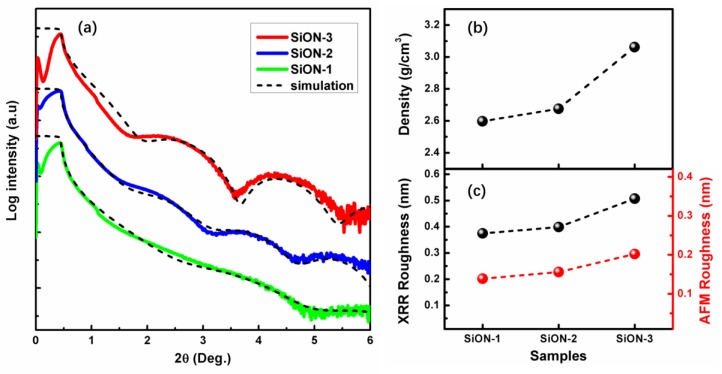
(**a**) Measured and simulated X-ray reflection (XRR) curves of three kinds of SiON film. (**b**) Density and (**c**) RMS roughness of these films obtained by XRR simulation.

Figure 2a shows the measured and simulated XRR curve of the SiON film with different oxygen composition. The XRR spectra is simulated with a bilayer composed of a thin SiO_2_ buffer and the SiON film. The simple two-layer model fits very well with the data of all the films. The total external reflection angles of the SiON-1 sample is ~0.44°, it has a slight rise and becomes ~0.45° for the SiON-2 sample, then it increases to ~0.49°, revealing that the mass density of these three films increases, as shown in Figure 2b. The density of SiON-1 sample is 2.6 ± 0.1 g/cm^3^, with a slightly larger than the density of SiO_2_ (2. 2 ± 0.1 g/cm^3^), which means the film only contains such little part of nitrogen that it almost becomes a pure SiO_2_ film. Then, the mass density becomes 2.7 ± 0.1 g/cm^3^ for the SiON-2 film. Furthermore, the value of mass density goes on increases to 3.1 ± 0.1 g/cm^3^ for SiON-3 film. This is very close to the density of silicon nitride (3.2 g/cm^3^) [33], which means the SiON-3 film contains an insignificant part of oxygen concentration and the film is mainly consisted as silicon nitride.

**Figure 3:**
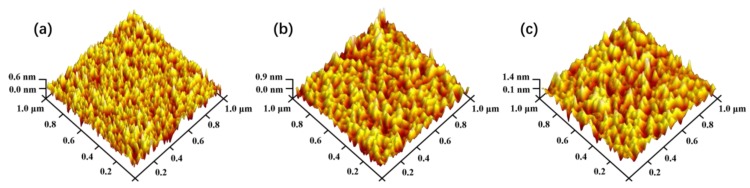
Three-dimensional morphology of the (**a**) SiON-1, (**b**) SiON-1, and (**c**) SiON-3 thin film by AFM.

The surface roughness obtained by XRR and AFM is shown in Figure 2c. The corresponding AFM mapping images of the films are shown in Figure 3. It is found that the surface roughness of all the films prepared in this work is very small, which means the film is very smooth. It should be noted here that both of XRR and AFM data present the similar trend of variation for roughness. Obviously, the RMS simulated from XRR data is almost two times higher than the value obtained by AFM, which is in agreement with the results reported in other work [34]. As see from the RMS values obtained by AFM, the SiON-3 film exhibits the highest RMS roughness value of about 0.2 nm, and the value of the other film is around 0.13–0.15 nm. 

### 3.2. Content and Composition Analysis

**Figure 4:**
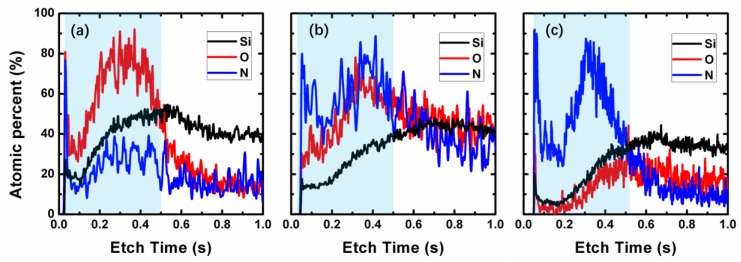
Glow discharge optical emission spectroscopy (GDOES) depth profile of Si, O, and N element in (**a**) SiON-1, (**b**) SiON-2, and (**c**) SiON-3 film.

To evaluate the distribution of Si, O, and N element in the entire film prepared in this work, all the samples were tested by GDOES firstly. Figure 4a–c show the GDOES depth profile of the SiON-1, SiON-2, and SiON-3 samples, respectively. The time needed for the etching of these three films is nearly the same, which means there little difference on the film thickness of these three samples. As shown in Figure 4, it is found that the average concentration of Si, O, and N element in the thin film (cyan region as be marked in Figure 4a–c) are all different for three samples. The concentration of oxygen is largest in SiON-1 sample but smallest in SiON-3 sample. However, the concentration of nitrogen presents completely the opposite variation tendency, which is smallest in SiON-1 sample but largest in SiON-3 sample. Then for SiON-2 sample, both of the oxygen and nitrogen concentration are kept medium. It is noteworthy that the variation trend of oxygen and nitrogen concentration in these samples verified the correctness of mass density obtained by XRR measurements. 

**Figure 5:**
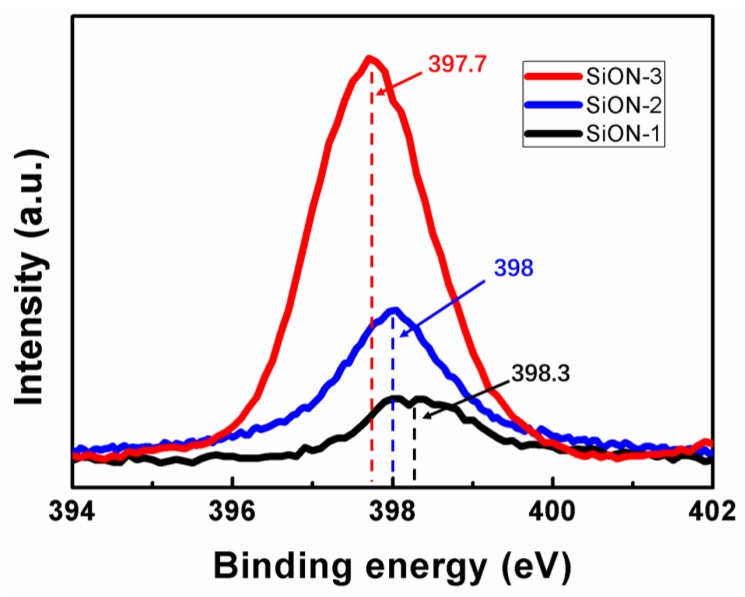
X-ray photoelectron spectra (XPS) of the studied samples in the energy region of N 1s levels.

X-ray photoelectron spectroscopy (XPS) was used to obtain detailed information about the elemental and chemical composition of the studied samples. Spectra of the as-prepared samples before the Ar^+^ ion bombardment contained only the characteristic peaks of photoelectrons created by the ionization of the inner electron shells of silicon (Si 2p) and oxygen (O 1s) atoms composing the SiO_2_ protective layer. To establish the composition of the SiON-1, SiON-2, and SiON-3 film under study, we applied the Ar^+^ ion treatment of the sample surface necessary for the removal of possible contamination and for etching of the protective SiO_2_ layer. Figure 5 displays the photoelectron spectra of the studied film in the energy region of N 1s levels. It can be seen clearly that the intensity of N1s peaks for SiON-1 film is weakest, SiON-2 film comes second, and SiON-3 film has the largest N 1s peak. This result means the content of nitrogen for SiON-3 film is much larger than SiON-1 and SiON-2 film. Besides, for SiON-1 film, the peak location of the N 1s is 398.3 eV, the value changed to 398 eV for SiON-2 film, and then it turned into 397.7 eV for SiON-3 film. From the variation of the location of N 1s peak, it can be found that the N 1s peak shifted toward higher energy when the content of oxygen increased in the SiON film. This can be explained by the redistribution of the electron density owing to the presence of oxygen in silicon oxynitride layers and by the presence of the contribution from the N–O bonds (e.g., (Si-)_2_ N–O with an energy of about 399.7 eV [35]).

**Figure 6:**
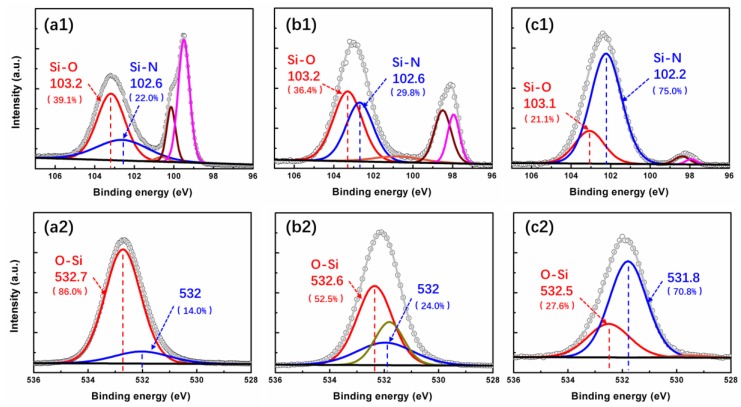
XPS analysis of SiON-1 (**a1**,**a2**), SiON-2 (**b1**,**b2**), and SiON-3 (**c1**,**c2**) sample on (**a1**,**b1**,**c1**) Si 2p spectra and (**a2**,**b2**,**c2**) O 1s spectra.

To further analyze the concentration, microstructure, chemical valence, and composition of the film and elements, the Si 2p and O 1s spectra of the SiON sample are examined by high-resolution XPS, as shown in Figure 6. From the Si 2p spectrum of the SiON-1 film (Figure 6a1), five peaks are observed at 103.2, 102.6, 100.6, 100.1, and 99.5 eV, corresponding to the Si–O (SiO_2_), Si–N (Si_3_N_4_), Si–N (SiN_x_), Si–Si (Si 2p 3/2), and Si–Si (Si 2p 1/2) bonds [36,37,38], respectively. The intensity and area of the Si–O related subpeak is larger than the Si–N ones, this result declared the SiON-1 film contains more content of oxygen than nitrogen. For the SiON-2 sample, three subpeaks centered at 103.3, 102.2, and 100.8 eV are observed except for the Si substrate. They are assigned to be the Si–O (SiO_2_), Si–N (Si_3_N_4_), and Si–N (SiN_x_) bonds, respectively. It is worth to point out that the intensity and area of the Si-N related subpeak increased compared with the strength in SiON-1 sample, which means the content of nitrogen increased in SiON-2 film. For SiON-3 sample, only two subpeaks centered at 103.2 and 102.2 eV are observed except for the Si substrate. They are assigned to be the Si–O (SiO_2_) and Si–N (Si_3_N_4_) bonds, respectively. It can be observed that the Si 2p spectra of the SiON-3 sample is dominated by the Si–N bonds. The existence of the Si–O bonds is ascribed to the oxidation effect after the Si_3_N_4_ film is exposed to the atmosphere. The other part of the Si–O bonds comes from the residual oxygen inside the film. By comparison of the O 1s spectra of SiON-1 (Figure 6a2) and SiON-2 samples (Figure 6b2) with that of the SiON-3 sample (Figure 6c2), more accurate bonding state information on the oxygen inside the SiON film can be achieved. All three films can be fitted mainly by two subpeaks located at ~532.6 and ~532 eV, corresponding to O–Si (bulk) and O–Si–N bonds, respectively [39]. For the O 1s spectra of the SiON sample, the existence of the O–Si bonds (Figure 6a2) corresponds with the Si–O bonds of the matching sample found in Si 2p (Figure 6a1). Moreover, the existence of the O–Si–N bonds confirms the formation of a ternary compound of SiO_x_N_y_ in the SiON sample. It reveals that the prepared SiON sample is not a “mixture” of Si_3_N_4_ and SiO_2_. Normally, ALD growth of ternary compound is realized by alternately growing two kinds of binary compounds. 

As the content of nitrogen plasma increasing from 95% to 99%, XPS intensity of the subpeak related to Si–O (SiO_2_) bonds decreases, while the subpeak centered around 532 eV (531.8 eV) increases. The variation of the intensity ratio of two subpeaks related to O–Si bonds indicates that the concentration of the oxygen decreases with the increasing of nitrogen plasma. The change of the O 1s and the Si 2p both implied that less Si–O bonds is present when increasing the nitrogen plasma, which agree with the previous researchers [35,36,37]. Besides, as seen in Figure 6, a small increase of the binding energy for Si–O, Si–N, and O–Si bonds is observed as increasing oxygen concentration. This small shift presents the same variation tendency with N 1s in Figure 5. It is well known that elements exhibit binding energy peaks, and their relative position depends on the electronegativity of surrounding atomic neighbors. Then, in the case of a phase mixture when more than one bonding state exists, a particular element is expected to have various binding energies, caused by different coordination/neighbors [36]. Therefore, the slight changes of binding energy for these chemical bonds in this study may also induced by the presence of oxygen in silicon oxynitride layers, and by the contribution from the O–N, Si–O–N, or other related bonds [35].

### 3.3. Optical Properties

In order to achieve in-depth knowledge on the evolution of the optical properties, variable angle (65°, 70°, and 75°) spectroscopic ellipsometry measurements have been performed on SiON film. The sample model used to analyze the raw ellipsometry data consists of (i) a semi-infinite crystalline silicon substrate, (ii) a native silicon dioxide layer, (iii) a homogenous, isotropic layer representing the SiON layer, (iv) surface roughness, and (v) air as the ambient medium. The top layer was described by an effective medium approximation (EMA) following the Bruggeman law [40] presents the surface roughness as a mix of the optical response of vacuum and SiO_2_ in a 50–50% concentration. The dielectric function of the SiON layer is modeled by the Tauc-Lorentz (T-L) oscillator model. The complex dielectric function of the energy (*E*) is defined as ε(E) = $\varepsilon_{1}$ + i$\varepsilon_{2}$. In the T-L model, $\varepsilon_{1}$ and $\varepsilon_{2}$ are defined as [41,42]:

(1) $$\varepsilon_{2}=\left[\frac{AE_{0}C{\left(E-E_{g}\right)}^{2}}{{\left(E^{2}-E_{0}^{2}\right)}^{2}+C^{2}E^{2}}\times \frac{1}{E}\right],\;E>E_{g},\;\varepsilon_{2}=0,\;E<E_{g},$$

and

(2) $$\;\varepsilon_{1}=\varepsilon_{1}\left(\infty \right)+\frac{2}{\pi }P\int_{E_{g}}^{\infty }\frac{\xi \varepsilon_{2}\left(\xi \right)}{\xi^{2}-E^{2}}d\xi$$

where *A*, *E_0_, C*, and *E_g_* represents the amplitude, peak transition energy, broadening term, and band-gap of the oscillator, respectively, all using units of energy (eV). For $\varepsilon_{1},\varepsilon_{1}\left(\infty \right)$ is a fitting constant to prevent $\varepsilon_{1}$ from converging to zero for energies below the band-gap, and *P* stands for the Cauchy principal part of the integral [42]. 

**Figure 7:**
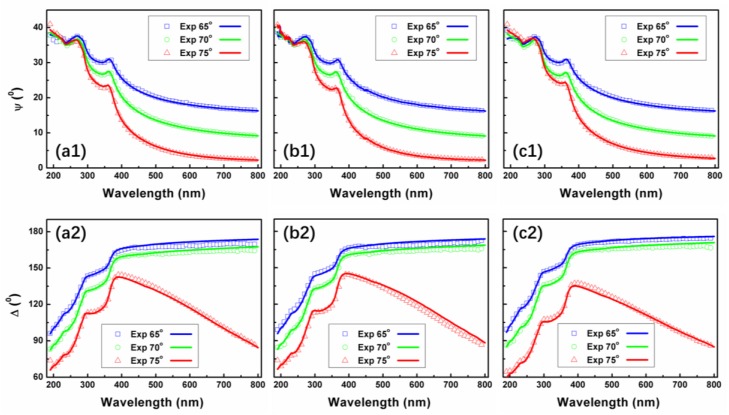
Ellipsometry measured and simulated Ψ (**a1**,**b1**,**c1**) and Δ (**a2**,**b2**,**c2**) for SiON samples for angles of incidence of 65°, 70°, and 75°.

SE measurements are performed at room temperature immediately after removing the samples from the deposition system to avoid surface contamination. The measured and simulated Ψ and Δ of SiON thin films for angles of incidence of 65°, 70°, and 75° are shown in Figure 7. The degree of fitting are all above 99% in the desired wavelength range (190–800 nm). As shown in Figure 7, the simulated data (solid line) are indistinguishable from the measured data (scatter line), indicating that the structure of the optical oscillator model used is unique below, through, and above the bandgap energy (i.e., UV–VIS–NIR). A remarkable fact in this figure is that both of Ψ and Δ for each sample decreased with increasing angles of incidence from 65° to 75° gradually. This difference could be related to the altered microstructure (surface morphology, roughness, and density) seen for these samples (Figure 2 and Figure 3) and points to the fundamental role this characteristic could play in the optical behavior of nanostructured SiON layers.

**Figure 8:**
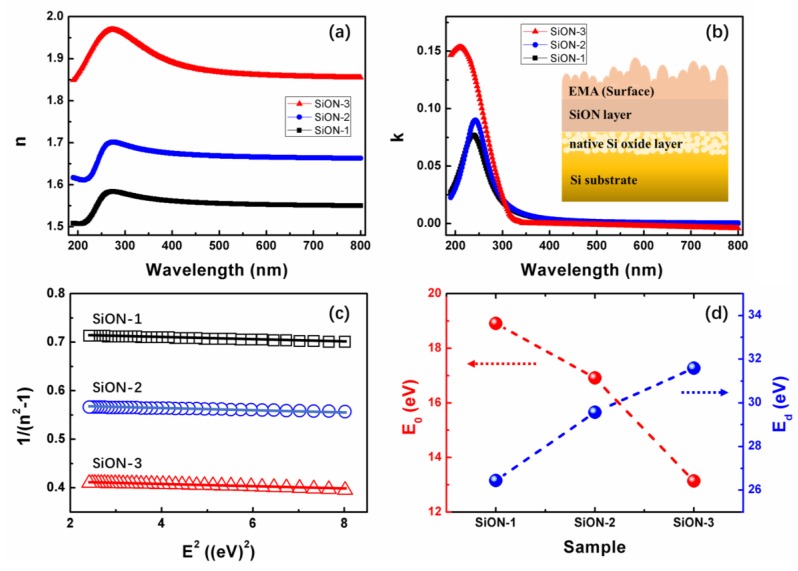
(**a**) Refractive index and (**b**) extinction coefficient of SiON thin film as a function of the wavelength. The inset in (**b**) displays the schematic representation of the layer structure employed to model the experimental data. (**c**) Dependence of 1/(*n*^2^ − 1) as a function of square photon energy obtained from SE (symbols) and linear fit of this data (solid lines) according to Wemple-DiDomenico model [43]. (**d**) Parameters *E*_0_ and *E*_d_ for different SiON film.

Basing on the above theory, the optical constants of SiON thin film are extracted and obtained from the fitted Ψ and Δ. Figure 6a shows the changes of the refractive index (*n*) as a function of wavelength in the range of 190–800 nm. Evidently, SiON-1 film exhibits the lowest n value of 1.55 at 632.8 nm, while the SiON-3 film exhibits a highest n value of 1.86 at 632.8 nm. Obviously, the refractive index decreases monotonously when oxygen content increases. It is well know that SiO_2_ film presents a low refractive index of about 1.4. As the oxygen content in SiON film increases, the ratio of SiO_2_ in the multilayer mixed with SiN_x_, SiO_2_, and other constituents will increase gradually, which, to some extent, induces the decrease of the refractive index of SiON. Figure 8b describes the extinction coefficient k of these three films. The inset presents the structure that is used to describe the SiON samples and model the ellipsometry data. The k value of all films is close to zero infinitely in the wavelength range of 400–800 nm, which indicates that the films are nearly transparent in this wavelength region [43]. Moreover, a red shift of the absorption edge can be observed with decreasing oxygen composition in SiON film, which is supposed to result from the variation of band-gap among these films, which will be discussed later. Therefore, the refractive index and the extinction coefficient of the SiON film can be effected by varying the oxygen concentration.

The refractive index dispersion *n* of SiON film was further fitted by the Wemple-DiDomenico model [44]. Then, the effect of oxygen concentration on the refractive index dispersion data in the spectra below the band-gap (1.55 eV < *E* < 2.8 eV) was investigated. The refractive index data can be fitted in this spectral range to the single oscillator expression [45]:

(3) $$n^{2}=1+\frac{E_{0}\cdot E_{d}}{E_{0}^{2}-E^{2}}\;$$

where *E* represents the photon energy, *E*_0_ is the oscillator energy, and *E*_d_ is called dispersion energy, which measures the oscillator strength (the strength of interband optical transitions). From linear regression of dependence (*n*^2^ − 1)^−1^ against *E*^2^ (as shown in Figure 8c), the parameters *E*_0_ and *E*_d_ can be calculated.

Figure 8d displays the variation of *E*_0_ and *E*_d_ of different SiON film. For SiON-1 sample, *E*_0_ is ~19 eV and *E*_d_ is ~26 eV. For SiON-2 sample, *E*_0_ is ~17 eV and *E*_d_ is ~30eV. Then, for SiON-3 sample, *E*_0_ is ~13 eV and *E*_d_ is ~32 eV. For the dispersion energy, an empirical relation is established [45]: *E*_d_ = *βN*_c_*N*_e_*Z*_a_, where *β* is a constant, according to Wemple, β has a value of 0.37~0.04 eV. *N*_c_ is the coordination number of the nearest neighboring cation to the anion, and *Z*_a_ is the formal chemical valency of the anion, then *N*_e_ is the total number of valence electrons per anion [46]. For Si_3_N_4_, *N*_e_ = [(4 valence electrons)·(3 silicon cation) + (3 valence electrons)·(4 oxygen anions)]/4 = 6 and *Z*_a_ = 3. For SiO_2_, *N*_e_ = [(4 valence electrons)·(1 silicon cation) + (6 valence electrons)·(2 oxygen anions)]/2 = 8, and *Z*_a_ = 2. Thus, *N*_c_ can be calculated from the relationship: *N*_c_ = *E*_d_/*βN*_e_ *Z*_a_. for SiON-1 sample, *N*_c_ ranges from 3.6 to 4.1, and it becomes 4.1 to 4.6 for SiON-2 sample, then for SiON-3 sample, it ranges from 4.3 to 4.9. From the variation trend of *E*_d_ and *N*_c_, it is found that the values of these two parameters increase with the decrease of oxygen concentration in SiON film. So, it can be concluded that SiON-1 film has a more amorphous structure than SiON-3 film. This result correlates with the fact that the film with more oxygen defects is less dense and have a lower refractive index [47]. Evidently, Figure 2b and Figure 8a demonstrate that the variation of film density and refractive index agree with the variation of *E*_d_ and *N*_c_ perfectly. Furthermore, the increase of *E*_d_ from SiON-1 to SiON-3 sample is also supposed to relate to the effective number of valence electrons, the variation of Si-N, Si-O, and Si-Si bonds, as shown in Figure 6.

Moreover, the long-wavelength limit of the refractive index, *n*(0), is given by [47]: *n*^2^(0) = 1+ *E*_d_/*E*_0_. Then *n*(0) is calculated to be 1.55, 1.66, and 1.85 for SiON-1, SiON-2, and SiON-3, respectively. These simulated results are in excellent agreement with the refractive index in Figure 8a, which improved the correctness of the model.

**Figure 9:**
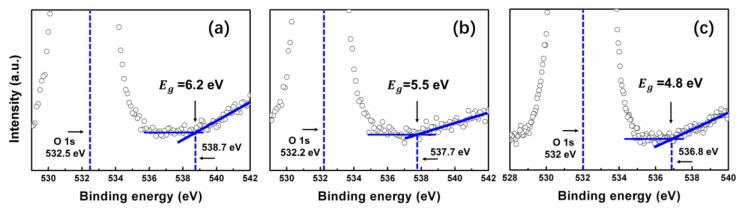
The determination of *E*_g_ through the O 1s peak analysis by XPS measurement for (**a**) SiON-1, (**b**) SiON-2, and (**c**) SiON-3 film.

It is important to determine the optical band-gap energy (*E*_g_) of a thin film material, because *E*_g_ is usually essential to develop the electronic band structure of a material, besides, *E*_g_ also effects the application of a film in the field of optoelectronic devices. Thus, we calculated the *E*_g_ of these SiON films. Figure 9 shows the determination of the *E*_g_ of the ALD SiON thin film using XPS O1s data. The method using the energy-loss peak of the O 1s spectrum to determine the *E*_g_ value of large band-gap materials has been reported by numerous scientists [48,49,50]. Thus, a representative high-resolution scan of the O 1s core level of SiON-1, SiON-2, and SiON-3 film obtained in this work are shown in Figure 9a–c. We can notice that the *E*_g_ is 6.2, 5.5, and 4.8 eV for SiON-1, SiON-2, and SiON-3 film, respectively. Which indicates that the optical band-gap energy of SiON films decreases with decreasing oxygen composition. 

### 3.4. Electrical Properties

**Figure 10:**
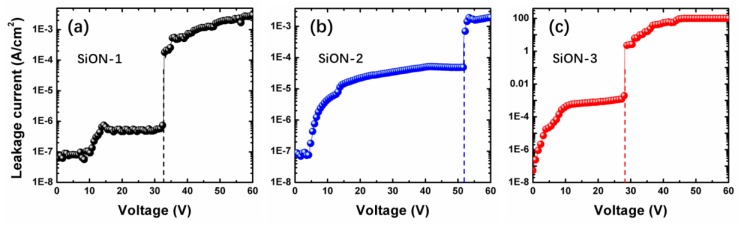
J−V curves of the (**a**) SiON-1, (**b**) SiON-2, and (**c**) SiON-3 dielectrics on Si substrate.

To examine the reliability of SiON film prepared in this work, J−V tests were performed on the prepared samples. The Cr/Au top electrodes (with a thickness of 200 nm and 50–300 μm in width square shape arrays) were deposited on the samples. As shown in Figure 10, all of the three samples exhibit no visible “soft” breakdown (lead by the defects in the dielectric film) point until the final “hard” (intrinsic) breakdown point is reached. This indicates that there are no obvious pinholes or cracks inside the dielectric film. Besides, the leakage current of all samples is about 1 × 10^−7^ A/cm^2^ at 1 V. For SiON-1 film, the leakage current keep at 1 × 10^−7^ A/cm^2^ until the voltage increases to 10 V. For SiON-2 film, the leakage current fluctuates around 1 × 10^−7^ A/cm^2^ until the voltage increases to 5 V. However, for SiON-3 film, the leakage current has a sharp increase at the very beginning when the voltage increases from 0 V to 5 V. These results suggest that the insulation characteristics of these three samples exhibit a big difference. In addition, we found the film presents lower leakage current and better insulativity with increasing oxygen concentration in SiON film, which makes it more suitable for dielectric and passivation materials [51]. As reported by many previous studies, the electrical insulation of SiO_2_ film is better than Si_3_N_4_ film [52,53]. It is suggested the SiON-1 sample with largest oxygen content has the best electrical insulation compared to other samples. As seen in Figure 10, the breakdown voltage is 33 V and 28 V for SiON-1 and SiON-3 film. However, the one for SiON-2 film is around 52 V, which is obviously much higher than other two samples. It is suggested that the breakdown field of SiON-2 sample is highest as the thickness of all three samples are kept the same (~15 nm). This result presents familiar tendency with AlON film in previous article [34], in which the AlON film shows higher breakdown field than both of Al_2_O_3_ and AlN film. The possible reason is that the nitrogen concentration in the SiON (AlON) film is beneficial for relieving the interfacial strain at the Si/SiON (Si/AlON) interface [54]. On the basis of this finding, the different characteristics of SiON film with different oxygen concentration can be utilized to fabricate high-performance electrical devices with desired properties.

## 4. Conclusions

In this study, high quality nano-scale SiON thin film was successfully deposited by using PEALD. The precursors of N_2_ and O_2_ are simultaneously introduced into the chamber during film growth at a temperature of 300 °C. It is believed that the composition of the obtained film quickly changes from SiN_x_ to SiO_2_ when a small amount of O_2_ is introduced into the chamber. From various measurements of SiON film with different oxygen concentration, the effects of oxygen concentration on properties of SiON film are systematically studied. It is found that the refractive index of SiON film increases from ~1.55 to ~1.86 as oxygen content decreases, while the surface roughness increases from 0.13 nm to 0.2 nm. Furthermore, the energy band-gap of SiON film decreases from ~6.2 eV to ~4.8 eV as the oxygen concentration in the film reduces. Regarding the electrical aspect, the MOS capacitor with SiON dielectric shows different electrical properties, the leakage current of SiON film appears inversely proportional to the oxygen concentration, and the SiON film with medium oxygen concentration presents much higher breakdown voltage than the other films. These results indicate that the oxygen concentration has an obvious influence on the microstructural, optical, and electrical properties of SiON film. In addition, various findings in this work inspire us to explore for further applications of SiON film in optoelectronic devices.
